# Krebs Cycle Intermediates Protective against Oxidative Stress by Modulating the Level of Reactive Oxygen Species in Neuronal HT22 Cells

**DOI:** 10.3390/antiox6010021

**Published:** 2017-03-16

**Authors:** Kenta Sawa, Takumi Uematsu, Yusuke Korenaga, Ryuya Hirasawa, Masatoshi Kikuchi, Kyohei Murata, Jian Zhang, Xiaoqing Gai, Kazuichi Sakamoto, Tomoyuki Koyama, Takumi Satoh

**Affiliations:** 1 Department of Anti-Aging Food Research, School of Bioscience and Biotechnology, Tokyo University of Technology, 1404-1 Katakura, Hachioji 192-0982, Japan; b01131253c@edu.teu.ac.jp (K.S.); b01130437a@edu.teu.ac.jp (T.U.); b0113111b5@edu.teu.ac.jp (Y.K.); b01132115c@edu.teu.ac.jp (R.H.); b01130888d@edu.teu.ac.jp (M.K.); b0113245f8@edu.teu.ac.jp (K.M.); 1099813793zj@gmail.com (J.Z.); g11160462a@edu.teu.ac.jp (X.G.); 2 Graduate School of Life and Environmental Sciences, University of Tsukuba, 1-1-1 Tennoudai, Tsukuba, Ibaraki 305-8572, Japan; sakamoto@biol.tsukuba.ac.jp; 3 Laboratory of Nutraceuticals and Functional Foods Science, Graduate School of Marine Science and Technology, Tokyo University of Marine Science and Technology, 4-5-7 Konan, Tokyo 108-8477, Japan; tskoyama@kaiyodai.ac.jp

**Keywords:** oxaloacetic acid, alpha-ketoglutaric acid, pyruvic acid, reactive oxygen species, neuron

## Abstract

Krebs cycle intermediates (KCIs) are reported to function as energy substrates in mitochondria and to exert antioxidants effects on the brain. The present study was designed to identify which KCIs are effective neuroprotective compounds against oxidative stress in neuronal cells. Here we found that pyruvate, oxaloacetate, and α-ketoglutarate, but not lactate, citrate, iso-citrate, succinate, fumarate, or malate, protected HT22 cells against hydrogen peroxide-mediated toxicity. These three intermediates reduced the production of hydrogen peroxide-activated reactive oxygen species, measured in terms of 2′,7′-dichlorofluorescein diacetate fluorescence. In contrast, none of the KCIs—used at 1 mM—protected against cell death induced by high concentrations of glutamate—another type of oxidative stress-induced neuronal cell death. Because these protective KCIs did not have any toxic effects (at least up to 10 mM), they have potential use for therapeutic intervention against chronic neurodegenerative diseases.

## 1. Introduction

The Krebs cycle is a series of enzymatic reactions that catalyze the aerobic metabolism of fuel molecules to carbon dioxide and water, thereby generating energy for the production of adenosine triphosphate molecules. Many types of fuel molecules can be drawn into and utilized by the cycle, including acetyl coenzyme A, derived from glycolysis or fatty acid oxidation. In eukaryotic cells, most of the enzymes catalyzing the reactions of the Krebs cycle are found in the mitochondrial matrixes [1]. The compounds involved in the cycle—termed Krebs cycle intermediates (KCIs)—function as energy donors and precursors for the synthesis of amino acids, lipids, and carbohydrates [2].

This aerobic metabolism may pervade every aspect of biology and medicine, because many papers published within the last 10 years suggest that KCIs regulate epigenetic processes and cellular signaling, possibly via protein binding [3]. KCIs activate specific signaling transduction pathways and exert various biological actions, such as neuroprotection, anti-inflammation, osteogenesis, and anti-aging [3]. For example, external supplementation with pyruvate (PA), oxaloacetate (OAA), α-ketoglutarate (AKG), malic acid (MA) or fumarate (FA), but not lactic acid (LA), succinate (SA), citrate (CA) or iso-citrate (ICA) significantly extends the lifespan of *Caenorhabditis elegans* by activating various transcriptional factor(s)-dependent transcriptional pathways [4,5,6]. Although fragmental information about the physiological roles of KCIs in the brain is available, KCIs are also proposed as being cardioprotective agents against myocardical infarction [7]. FA and AKG have been proposed to protect cardiac muscles possibly through activating or inhibiting specific transcription factors, such as NF-E2 related factor 2 (NRF2) and hypoxia inducible factor (HIF-1), respectively. In neurons, PA prevents hydrogen peroxide (H_2_O_2_)-induced cell death [8,9], protects the brain against experimental stroke via an anti-inflammatory mechanism [10], and prevents the age-dependent cognitive deficits seen in a mouse model of Alzheimer’s disease [11]. These protections are possibly due to its α-ketoacid structure, which can directly react with H_2_O_2_ [8,9].

H_2_O_2_ is a stable, uncharged, and freely diffusible reactive oxygen species (ROS) with a putative second messenger role in intracellular and extracellular signaling mechanisms [12]. The generation of H_2_O_2_ is relatively high in the brain, partly because of the high activities of oxygen consumption and partly because of the high level of expression of monoamine oxidase in this tissue [13]. In pathological conditions such as ischemia–reperfusion and Alzheimer’s disease, various cell types may produce large amounts of H_2_O_2_ [13]. In addition to enzymatic defense mainly mediated by various enzymes (i.e., glutathione (GSH) peroxidase, catalase, and peroxiredoxins), non-enzymatic mechanisms can also contribute to the cellular defense against H_2_O_2_-induced cytotoxicity [13]. For instance, PA is abundant in mammalian cells and has the property of non-enzymatically reacting with H_2_O_2_ [8,9]. PA protects neurons against both exogenous and endogenous H_2_O_2_, and it also inhibits cell death mediated by H_2_O_2_ in neurons [8] and non-neuronal cells [9]. However, whether KCIs other than PA, OAA, and AKG really elicit neuroprotection through a simple direct interaction with exogenous H_2_O_2_ at extracellular locations has not yet been fully clarified. 

In this present study, we examined the neuroprotective effects of KCIs against two types of oxidative stress in neuronal HT22 cells and found that only three of them (PA, OAA, and AKG)—which have an α-keto acid structure—had significant neuroprotective effects by modulating the levels of ROS in the cells. We found that the other KCIs were not protective, suggesting that these neuroprotective KCIs—used singly or as a cocktail—could be a potential food supplement for the purpose of preventing chronic neurodegeneration.

## 2. Materials and Methods

### 2.1. Chemicals

All KCIs (sodium salt), sodium glutamate (Glu), hydrogen peroxide (H_2_O_2_), and 3-(4,5-dimethylthiazol-2-yl)-2,5-diphenyl tetrazolium bromide (MTT) were purchased from Wako Junyaku (Tokyo, Japan). Stock solutions of KCIs (100 or 1000 mM) were prepared in Ca^2+^, Mg^2+^-free phosphate-buffered saline (PBS(-), Invitrogen, Carlsbad, CA, USA). 2′,7′-Dichlorodihydrofluorescin diacetate (DCFH-DA, Sigma-Aldrich, St. Louis, MO, USA) stock solution was prepared in 10 mM dimethyl sulfoxide solution and used at 10 μM in the culture medium.

### 2.2. HT22 Cultures and MTT Assay

HT22 hippocampal neuronal cells were cultured as described previously [14,15,16]. In HT22 cells, high concentrations (mM levels) of Glu can induce cell death by depleting intracellular GSH through inhibition of cystine influx, and relatively low concentrations (μM levels) of H_2_O_2_ can induce cell death. Because HT22 cells do not have functional NMDA receptor proteins, they do not die due to excitotoxicity [14,15,16]. These cells were maintained in 10-cm dishes (Invitrogen, Carlsbad, CA, USA) containing 10 mL of Dulbecco’s Modified Eagle medium supplemented with 10% (*v*/*v*) heat-inactivated (56 °C, 30 min) fetal calf serum (Invitrogen). The cells were seeded into 24-well plates at a density of 4 × 10^4^ cells/cm^2^. When examining their effects on the H_2_O_2_ toxicity, we added KCIs to the cultures after a 24-h incubation. Sixty minutes later, 100, 200, or 500 μM H_2_O_2_ was added, and the cells were then incubated for an additional 24 h. We set differential pre-incubation time 24 h and 1 h for Glu- and H_2_O_2_-toxicity, respectively. This was to stabilize the response to H_2_O_2_, and also because the Glu toxicity is completely inhibited by the long preincubation (over 5 h) by an unknown mechanism [14,15,16]. When the effects of KCIs on oxidative Glu toxicity were examined, the cells were incubated for 1 h after having been seeded in wells of a 24-well plate, and the KCIs were then added to the cultures. Sixty minutes later, 5, 10, or 20 mM Glu was added, and the cells were then incubated for an additional 24 h. To evaluate survival of the HT22 cells, we performed the MTT assay [14,15,16].

### 2.3. DCF Assay

The extent of cellular oxidative stress was assessed by monitoring the formation of free-radical species by using DCFH-DA, as described elsewhere [17]. Cells were plated 24 h before initiation of the experiment at a density of 40,000 cells/well in 24-well plates. KCIs and 10 μM DCFH-DA were added to the cells 30 min before the measurement. The plate was set into a Spark10M (Tecan Japan, Tokyo, Japan) under an atmosphere of 5% CO_2_ and temperature of 37 °C. H_2_O_2_ (200 μM) or vehicle was added to wells at 30 min, and the cells were incubated further for 180 min. DCF fluorescence was measured at a 485-nm excitation wavelength and 538-nm emission wavelength at 10-min intervals. Fluorescence values were expressed as a percentage of the value for the untreated control. 

### 2.4. Statistical Analysis

Experiments presented herein were repeated at least three times, with each experiment performed in quadruplicate. Data were presented as the mean ± SD. The statistical significance of differences was examined by performing Student’s *t*-test.

## 3. Results

### 3.1. Neuroprotective Effect of PA, OAA, and AKG on HT22 Cells

**Figure 1:**
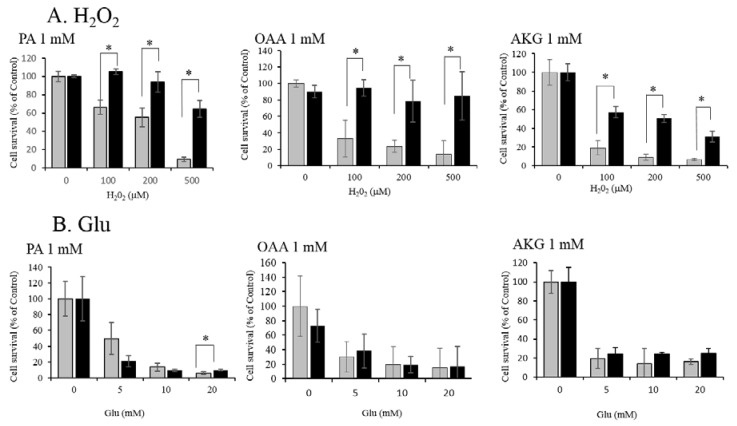
Protection of HT22 against (**A**) H_2_O_2_ and (**B**) Glu by pyruvate (PA), oxaloacetate (OAA), and α-ketoglutarate (AKG). (**A**) Protective effects of 1mM PA, OAA, or AKG against various concentrations of H_2_O_2_. (**B**) No protective effect of 1 mM PA, OAA, or AKG against high concentrations of Glu was found. White bars indicate H_2_O_2_ or Glu alone; and gray bars H_2_O_2_ + KCI (PA, OAA, or AKG). Values, presented as a percentage of the control MTT (3-(4,5-dimethylthiazol-2-yl)-2,5-diphenyl tetrazolium bromide) value (obtained in the absence of glutamate), are given as the mean ± SD (*n* = 4). * significantly different (*p* < 0.05) from samples without a KCI. KCI: Krebs cycle intermediate.

**Figure 2:**
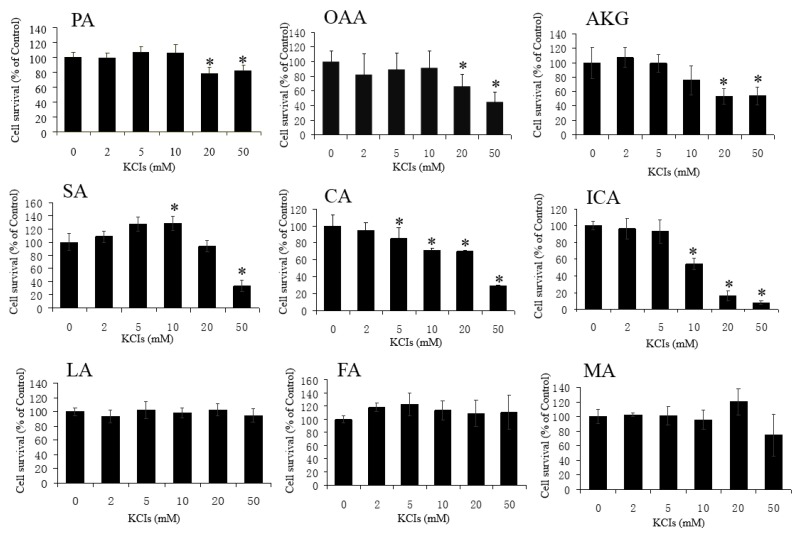
Toxic effects of KCIs by themselves. PA, OAA, AKG, succinate (SA), lactic acid (LA), fumarate (FA), and malic acid (MA) did not reduce cell survival up to 10 mM, whereas citrate (CA) and iso-citrate (ICA) reduced it when used at concentrations around 5–10 mM. Values, presented as a percentage of the control MTT value (obtained in the absence of glutamate), are given as the mean ± SD (*n* = 4). * significantly different (*p* < 0.05) from samples without a KCI.

We used HT22 cells—a neuronal cell line derived from a mouse hippocampus—as a model for oxidative cell damage. Treatment of the cells with 100, 200, or 500 μM H_2_O_2_ (Figure 1A) or with 5, 10, or 20 mM Glu (Figure 1B) induced cell death by oxidative stress within 24 h. Pre (1 h)-treatment of the cells with the KCIs at 1 mM protected the cells from the toxic effects of H_2_O_2_ (Figure 1A), whereas these KCIs were not—or very little if they were—protective against Glu toxicity (Figure 1B). These KCIs were not toxic to HT22 cells up to 10 mM, but they caused a small but significant reduction in cell survival when used at 20–50 mM (Figure 2). These results suggest that PA, OAA, and AKG could protect neuronal cells against H_2_O_2_, but not against Glu.

### 3.2. Regulation of ROS by PA, OAA, and AKG

**Figure 3:**
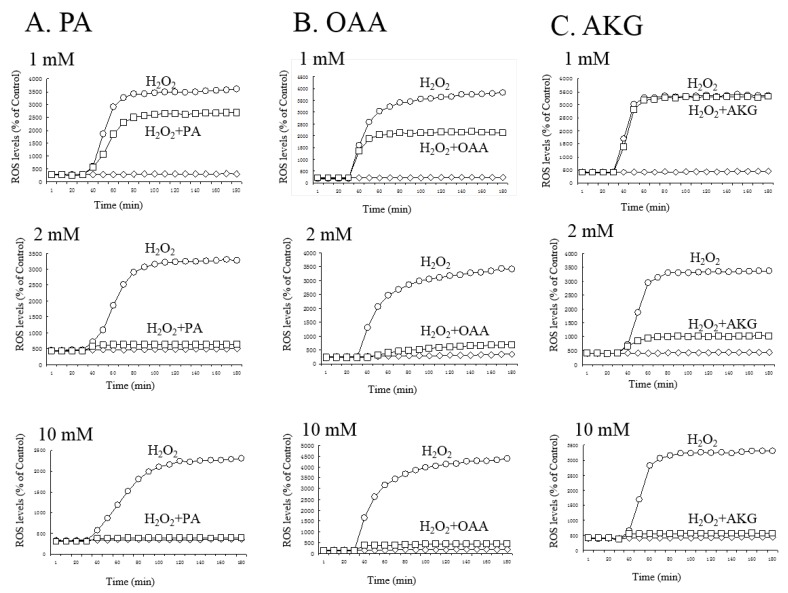
Modulation of reactive oxygen species (ROS) level by (**A**) PA, (**B**) OAA, or (**C**) AKG against H_2_O_2_-induced cytotoxicity. The measurement of the cells loaded with 10 μM DCFH-DA (2′,7′-Dichlorodihydrofluorescin diacetate) for 30 min started at 0 min, and DCF fluorescence levels are shown at 10-min intervals. Various concentrations (1, 2, and 10 mM) of PA, OAA, or AKG were added to the cultures at the same time as DCFH-DA addition. H_2_O_2_ (200 μM) was added at 30 min. Values are the means ± SD from four experiments per group. Diamonds, control; circles, H_2_O_2_; squares, H_2_O_2_ + KCI. Note that the KCI alone groups were not shown in these graphs, because KCIs themselves did not affect ROS levels at any time point.

As the neuroprotective effects of the KCIs may have been due to the chemical structure of α-keto acid by direct interaction with H_2_O_2_ inside the cells, we next examined whether the KCIs could reduce the level of ROS in HT22 cells (Figure 3). We quantified the levels of ROS with the ROS-sensitive fluorescent indicator DCFH-DA by use of a Spark 10M device (Tecan Japan, Tokyo, Japan). Based on the fluorescence activity, H_2_O_2_ (200 μM) increased the levels of ROS by 20–40-fold. ROS levels plateaued at 45–60 min of exposure. PA (Figure 3A) or OAA (Figure 3B) at 1, 2, or 10 mM, and 2 or 10 mM AKG (Figure 3C) significantly lowered intracellular ROS formation; 1 mM AKG did not reduce the ROS level. These results suggest that these KCIs used at low mM levels effectively reduced the ROS level in neural cells. 

### 3.3. No Protective Effects by LA, CA, ICA, SA, FA, and MA against H_2_O_2_

**Figure 4:**
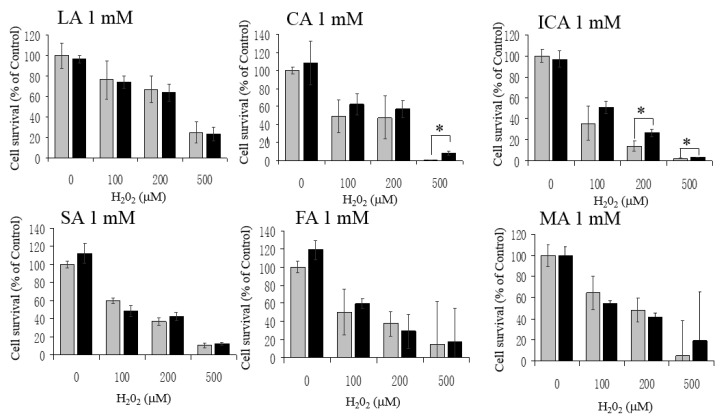
No protection of HT22 cells against H_2_O_2_ by the other KCIs at 1 mM. Neither LA, CA, ICA, SA, FA, nor MA protected the cells against H_2_O_2_-mediated cytotoxicity. White bars are H_2_O_2_ alone; and gray bars are H_2_O_2_ + KCI. Values, presented as a percentage of the control MTT value (obtained in the absence of glutamate), are given as the mean ± SD (*n* = 4). * significantly different (*p* < 0.05) from samples without a KCI.

**Figure 5:**
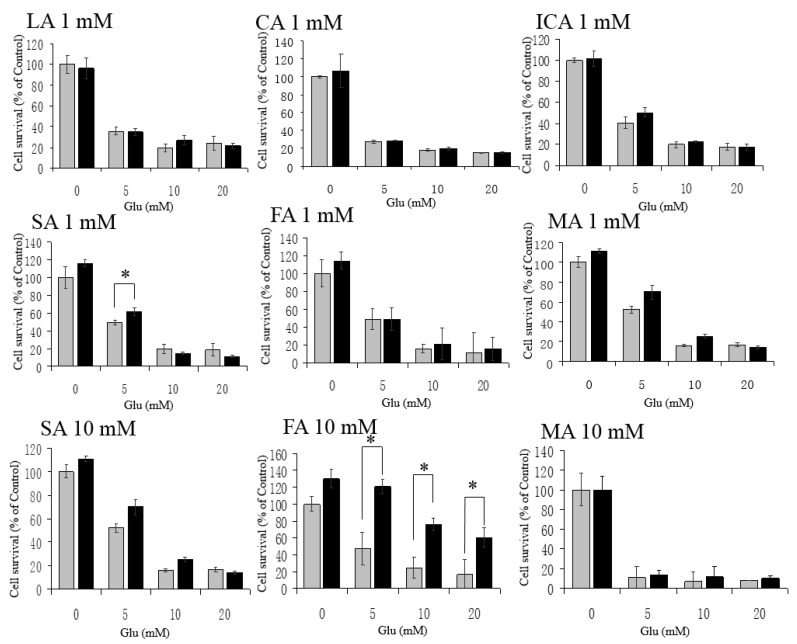
No protection of HT22 cells against Glu by the other KCIs at 1 mM. Neither LA, CA, ICA, SA, nor MA protected the cells against Glu used at 1 or 10 mM. FA at 10 mM protected the cells against Glu, although 1 mM FA did not. White bars indicate Glu alone, and gray bars Glu + KCI. Values, presented as a percentage of the control MTT value (obtained in the absence of glutamate), are given as the mean + SD (*n* = 4). * significantly different (*p* < 0.05) from samples without a KCI.

Next, we assessed whether LA, CA, ICA, SA, FA, and MA could protect HT22 cells against H_2_O_2_, and found that these intermediates were not—or were very little if they were—protective against H_2_O_2_ (100, 200, and 500 μM)-mediated cytotoxicity (Figure 4). Then, we examined whether these KCIs could protect the cells against Glu toxicity. Additionally, except for SA against 5 mM Glu, these KCIs (at 1 mM) showed no protection against high concentrations (5, 10, and 20 mM) of Glu (Figure 5). These results indicate that LA, CA, ICA, SA, FA, and MA could not protect HT22 cells against oxidative stress. LA, SA, FA, and MA were not toxic to the cells up to 10 mM. By some unknown mechanism, CA and ICA gave toxic effects when tested around 5–10 mM (Figure 2). A high concentration of FA had an exceptional action on the cells, because 10 mM FA gave significant protection against high concentrations of Glu (Figure 5).

## 4. Discussion

**Figure 6:**
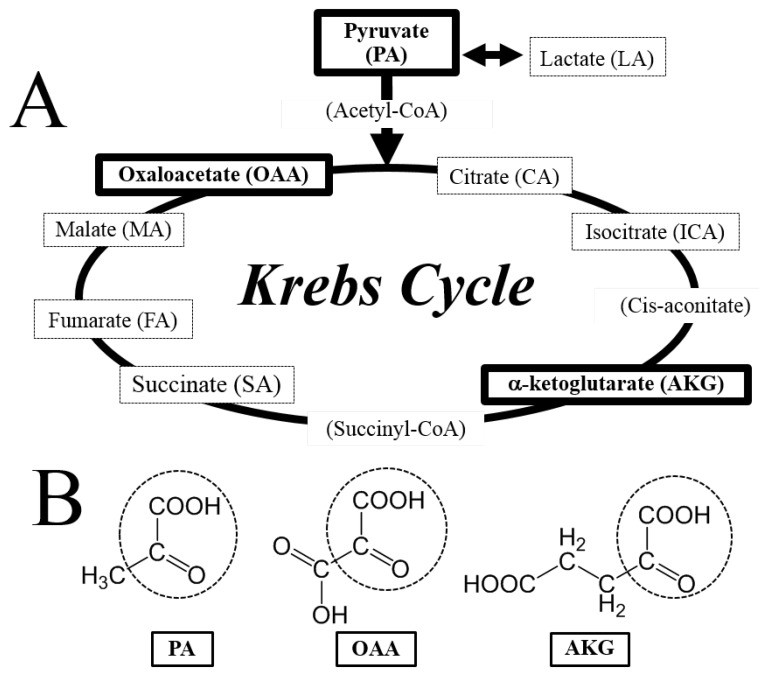
(**A**) Compounds involved in the Krebs cycle and (**B)** common chemical structure of neuroprotective KCIs (PA, OAA, and AKG). Note that PA, OAA, and AKG have the common chemical structure of the α-keto acid group—indicated by the dotted circle—with which molecules can directly react with H_2_O_2_.

Here, we found that the α-keto acid group-containing KCIs (PA, OAA, and AKG) could protect neurons against H_2_O_2_, possibly through direct interaction with H_2_O_2_, although we did not provide direct evidence for this chemical reaction itself. Because DCFH-DA is hydrolyzed by cytosolic esterase and is activated by ROS in the cytoplasm, the inhibition of ROS increase by PA, OAA, and AKG could be due to an H_2_O_2_-scavenging effect of them inside the cells. The conclusion of this study is illustrated in Figure 6A and B. PA, OAA, and AKG were neuroprotective, but not the other KCIs. For example, LA and MA— putative neuronal energy substrates that may produce protective KCIs through their metabolism [1,2]—did not protect the cells (Figure 4 and Figure 5). AKG may have other protective effects than α-keto acids such as PA and OAA. AKG at 1mM protected the cells against H_2_O_2_ (Figure 1) without causing a significant reduction in ROS levels (Figure 3). Because AKG is reported to activate the degradation of HIF-1α subunit and reduce the expression of downstream enzymes [6,7], reduction of this pathway may have been involved in the protective effects of AKG. FA may be another exceptional KCI. FA at 10 mM protected the cells against Glu-mediated cytotoxicity (Figure 5), although the other KCIs did not do so. Because FA is reported to activate the Nrf2 pathway and induce phase-2 enzymes [18], the activation of this pathway may have been involved in the FA-induced protection.

Interestingly, the protection afforded by PA, OAA, and AKG was totally different than that of NRF2 activators such as carnosic acid [19,20], zonarol [21,22], and tert-butyl hydroquinone [23]. These activators can protect cells against a high concentration of Glu, but not against H_2_O_2_. Nrf2 activators protect cells against high Glu concentrations by inhibiting the decrease in GSH by induction of Phase-2 enzymes, the action of which enhances the production of GSH [24,25,26]. The increase in GSH by NRF2 activators is not sufficient for protection against H_2_O_2_ [24,25,26]. 

## 5. Conclusions

This present study importantly suggests that there are 2 distinctive oxidative mechanisms: one induced by H_2_O_2_ and the other by Glu. α-Ketoacid group-containing KCIs (PA, OAA, and AKG) protected the cells against the former but not against the latter, whereas Nrf2 activators act vice versa. The central nervous system is particularly vulnerable to oxidative damage due to its high energy expenditure and oxygen demand [24,25,26]. Elevated concentrations of free radicals and resultant oxidative damage, such as lipid peroxidation and protein carbonylation, have been repeatedly demonstrated in neurodegenerative disorders such as Alzheimer’s disease, Parkinson’s disease, and ischemic stroke [24,25,26]. Thus, PA, OAA, and AKG, being natural metabolic intermediates and energy substrates, exert antioxidant effects in the brain and other tissues susceptible to H_2_O_2_.

## Abbreviations

AKG, α-ketoglutarate; CA, citrate; Cont, control; DCFH-DA, 2′,7′-Dichlorodihydrofluorescin diacetate; FA, fumarate; Glu, glutamate; GSH, glutathione; H_2_O_2_, hydrogen peroxide; HIF-1, hypoxia inducible factor; ICA, iso-citrate; KCI, Krebs cycle intermediate; LA, lactate; MA, malate; MTT, 3-(4,5-dimethylthiazol-2-yl)-2,5-diphenyl tetrazolium bromide; *NRF2*, NF-E2 related factor 2; OAA, oxaloacetate; PA, pyruvate; ROS, reactive oxygen species; PBS(-), Ca^2+^, Mg^2+^-free phosphate-buffered saline; SA, succinate; SD, standard deviation.
